# Bioenergetic Consequences of PINK1 Mutations in Parkinson Disease

**DOI:** 10.1371/journal.pone.0025622

**Published:** 2011-10-17

**Authors:** Andrey Yurevich Abramov, Matthew Gegg, Anne Grunewald, Nicholas William Wood, Christine Klein, Anthony Henry Vernon Schapira

**Affiliations:** 1 Department of Molecular Neuroscience, UCL Institute of Neurology, London, United Kingdom; 2 Department of Clinical Neurosciences, UCL Institute of Neurology, London, United Kingdom; 3 Department of Neurology, University of Luebeck, Luebeck, Germany; National Institute on Aging Intramural Research Program, United States of America

## Abstract

**Background:**

Mutations of the gene for PTEN-induced kinase 1 (*PINK1*) are a cause of familial Parkinson's disease (PD). PINK1 protein has been localised to mitochondria and *PINK1* gene knockout models exhibit abnormal mitochondrial function. The purpose of this study was to determine whether cells derived from PD patients with a range of *PINK1* mutations demonstrate similar defects of mitochondrial function, whether the nature and severity of the abnormalities vary between mutations and correlate with clinical features.

**Methodology:**

We investigated mitochondrial bioenergetics in live fibroblasts from *PINK1* mutation patients using single cell techniques. We found that fibroblasts from *PINK1* mutation patients had significant defects of bioenergetics including reduced mitochondrial membrane potential, altered redox state, a respiratory deficiency that was determined by substrate availability, and enhanced sensitivity to calcium stimulation and associated mitochondrial permeability pore opening. There was an increase in the basal rate of free radical production in the mutant cells. The pattern and severity of abnormality varied between different mutations, and the less severe defects in these cells were associated with later age of onset of PD.

**Conclusions:**

The results provide insight into the molecular pathology of *PINK1* mutations in PD and also confirm the critical role of substrate availability in determining the biochemical phenotype – thereby offering the potential for novel therapeutic strategies to circumvent these abnormalities.

## Introduction

Mutations in the gene for *PTEN-induced kinase 1* (*PINK1*) are a cause of autosomal recessive familial Parkinson's disease (PD) [1]. The clinical phenotype of *PINK1* mutant PD patients is often indistinguishable from idiopathic, sporadic PD [2], [3]. Thus the mechanisms by which mutations in this gene can induce dopaminergic cell death are a major focus of interest for those seeking to define the molecular pathogenesis of PD.

The function of the PINK1 protein is not yet defined, although it is known to be targeted to mitochondria [1], a significant component of PD pathogenesis [4], [5] and is thought to be involved in protection against free radical generation [6]. *PINK1* gene mutations or *PINK1* silencing result in reduced mtDNA levels, defective ATP production, impaired mitochondrial calcium handling, and increased free radical generation, which in turn result in a fall in mitochondrial membrane potential and an increased susceptibility to apoptosis in neuronal cells, animal models and patient-derived fibroblasts [7]–[12]. Recent studies have also demonstrated that PINK1 can initiate the translocation of parkin to mitochondria and the induction of mitophagy [13], [14]. Overexpression of parkin protein can rescue the effects of a *PINK1* mutation in *Drosophila* and mammalian cells again suggesting that these two proteins participate in related pathways [15]–[20].

Many of the studies performed to date to define the role of PINK1 have involved artificial cell models with overexpression of wild-type or mutant PINK1, or knock out in cell or animal models, and few have used endogenous expression of mutant protein in host cells. We have previously published on the biochemical effects of mutant *PINK1* expression in PD patient fibroblasts [11]. We have now investigated at the level of the single cell, the bio-energetic effects of endogenously expressed *PINK1* mutations in PD cells and demonstrate that the consequences depend upon the specific underlying mutation.

## Results and Discussion

**Figure 1:**
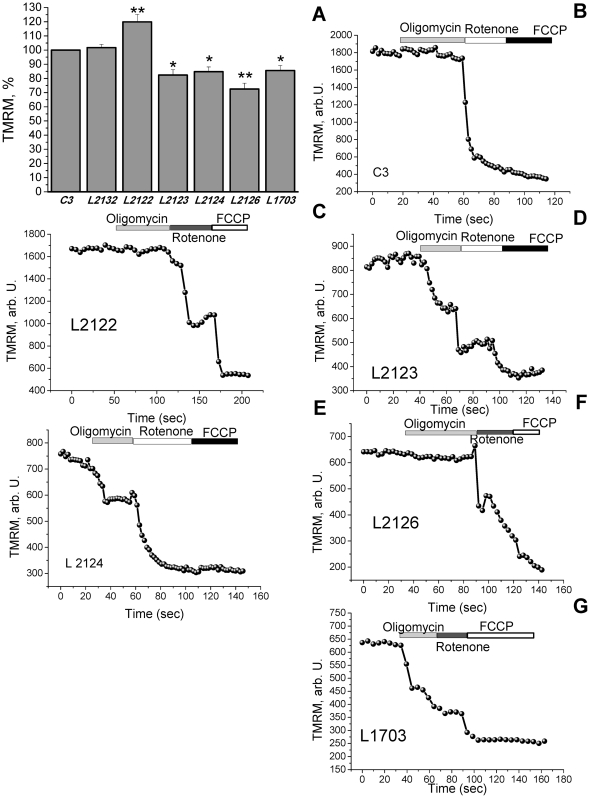
Characteristics of mitochondrial membrane potential (Δψ_m_) in human fibroblasts with PINK1 mutations. A-L2122 fibroblasts exhibited a 20% increase (p<0.001) in TMRM fluorescence (i,e, an increased Δψ_m_) compared to controls. Fibroblasts with PINK1 mutations (L2123, L2124, L2126 amd L1703) showed a significant decrease in Δψ_m_ compared to control cells. B–G In control, L2122 and L2126 fibroblasts (B–C, F), oligomycin did not affect Δψ_m_; rotenone induced a partial depolarisation; FCCP induced complete depolarisation. In L2123, L2124 and L1703 fibroblasts (D–E, G), oligomycin caused a mitochondrial depolarisation.

Four human fibroblasts with PINK1 mutations - L2123, L2124, L2126 and L1703 had a significant reduction of mitochondrial membrane potential (Δψ_m_) by 14–27%, with the maximal decrease in L2126 carrying the 1366C>T mutation by 27.5±2.1% of control cells (p<0.05; n = 4 experiments; Figure 1A). However, one mutant line, L2122 carrying the same 1366C>T mutation, showed a significantly increased Δψ_m_ of 119.9±5.3% (n = 68 cells; n<0.001) relative to the control cell lines C3 and L2132 (Figure 1A).

To investigate how mutations in PINK1 can induce different effects on the value of Δψ_m_, we explored the roles of different mitochondrial mechanisms important in the maintenance of membrane potential. In cells with normal oxidative phosphorylation, Δψ_m_ is maintained by the proton pumping activity of the respiratory chain. However if oxidative phosphorylation is impaired, the F_1_F_O_-ATP synthase (complex V) can reverse, hydrolyse ATP and pump protons across the inner membrane to maintain Δψ_m_ [21]. Substrate deprivation, such as has been described in PINK1 deficient models, can lead to a similar effect i.e. reversal of complex V [7]. Control cells, C3 (n = 41) and L2132 (n = 16), showed either no response (or a small hyperpolarisation) in response to complex V inhibition by oligomycin (0.2 µg/ml), while subsequent inhibition of complex I by rotenone (5 µM) caused a rapid loss of potential (Figure 1B). This confirms that in human fibroblast cells, Δψ_m_ is mainly maintained by respiratory chain function, and that in our system there is no limit to substrate availability.

A similar pattern of oligomycin resistance was seen in the L2122 (n = 31) and L2126 (n = 51) 1366C>T cell lines, which had the highest and lowest resting Δψ_m_respectively (Figure 1 C and F). In contrast, but in agreement with PINK1 deficient cell models [7], [22], oligomycin caused marked mitochondrial depolarisation in L2123 (by 37.6±3.1%, n = 33; figure 1D), L2124 (by 42.1±3.3%; n = 38; figure 1E) and L1703 (by 38.7±2.1%, n = 27; figure 1G). TMRM fluorescence was then significantly reduced in all cell lines by the subsequent addition of rotenone (note almost complete depolarisation in control and L2124 cells). A relatively large further decrease in signal in response to FCCP in L2122 then suggested that complex II activity must also be relatively active as a donor of electrons in these cells.

**Figure 2:**
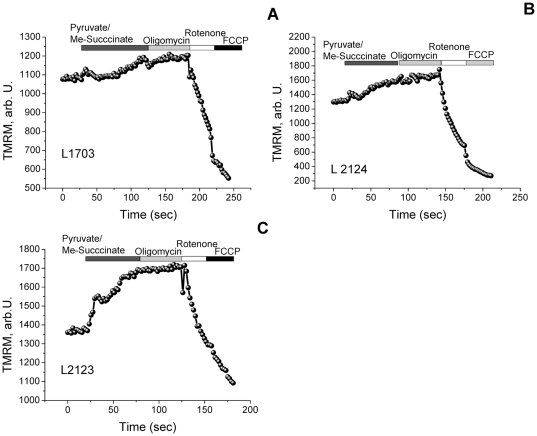
Effect of mitochondrial substrates on mechanism of maintenance of Δψ_m_ in human fibroblast with PINK1 mutations. Application of pyruvate (5 mM) or/and methyl succinate (5 mM) to fibroblasts increased Δψ_m_; Substrate provision prevented the oligomycin induced mitochondrial depolarisation in L2123, L2124 and L1703 fibroblasts.

Provision of additional substrate for complex I (pyruvate/malate) or complex II (methyl succinate) to PINK1 deficient neurons caused an increase in Δψ_m_ and restoration of normal maintenance of Δψ_m_ [7]. To investigate if a mutation in PINK1 would induce the same effect on mitochondrial substrate delivery, we explored the effect of mitochondrial substrates on the maintenance of Δψ_m_ in those cell lines that showed depolarisation in response to oligomycin – L2123, L2124 and L1703. Provision of additional substrates for complex I and II by application of 5 mM pyruvate or/and 5 mM methyl succinate to the media increased Δψ_m_ and completely prevented oligomycin-induced mitochondrial depolarisation in 2123 (n = 24), L2124 (n = 28) and L1703 (n = 38) cells (figure 2 A–C).

### Mitochondrial redox state

Given our demonstration above that cells with PINK1 mutations are dependent on the availability of respiratory chain substrates, we measured the autofluorescence of NADH and FAD+ as a function of respiratory chain activity and substrate turnover.

**Figure 3:**
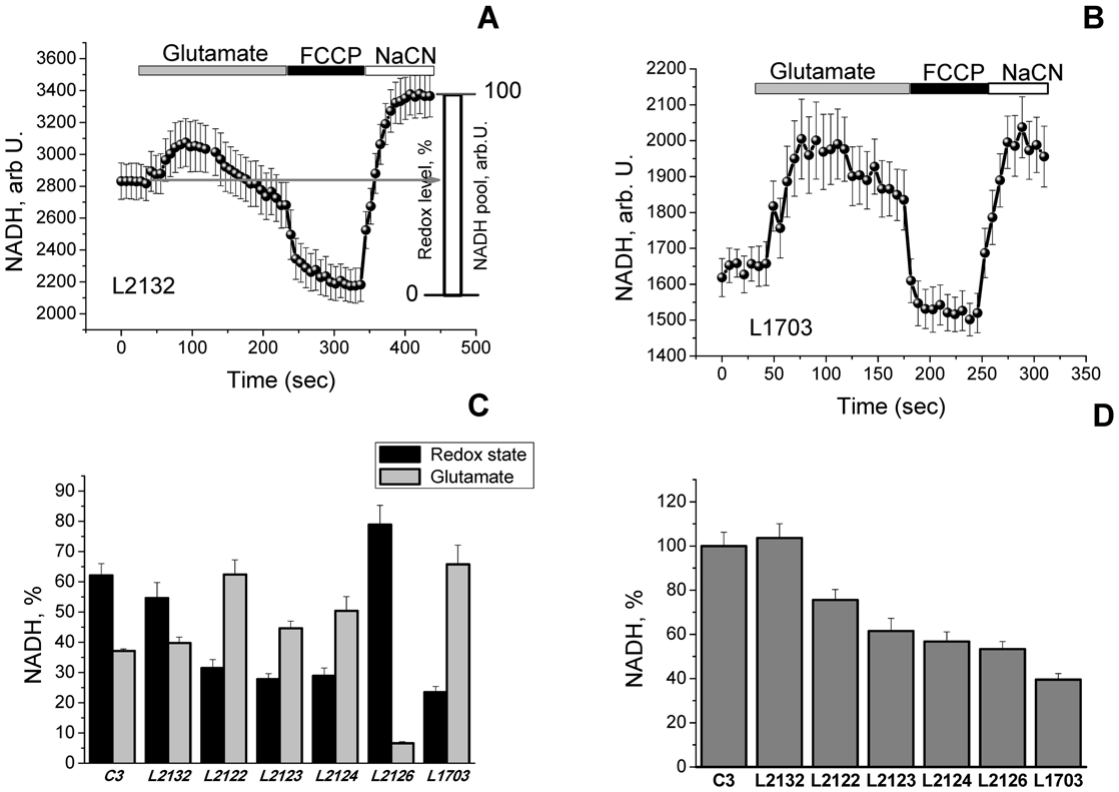
Redox state and NADH level in fibroblasts with PINK1 mutations and control cells. A–B Graphs demonstrate averaged trace of NADH autofluorescence in control L2132 fibroblasts (A) and L1703 PINK1 mutation (B). Estimation of the %age change in mitochondrial redox level in control cells – L2132 (A) and cells with mutations in PINK1 (B–C). C-Redox state was estimated as: 0 is response to FCCP (maximal rate of respiration and lowest level of mitochondrial NADH) and 100% is response to cyanide (inhibition of respiration with no consumption of NADH in mitochondria; Fibroblasts with PINK1 mutations have lower NADH redox state compared to control fibroblasts (except L2126) that can be normalized by application of 5 mM glutamate (C). D – Total pool of mitochondrial NADH was also significantly lower in fibroblasts with PINK1 mutations.

In these experiments, the resting level of NADH autofluorescence in the cells was expressed as a ‘redox index’, a function of the maximally oxidized and maximally reduced signals. These were estimated from the response to 1 µM FCCP (which stimulates maximal respiration, completely oxidizing the mitochondrial NADH pool) which was taken as 0%, and the response to 1 mM NaCN (which inhibits respiration, preventing NADH oxidation, and so promoting maximal NADH reduction and maximal autofluorescence) which was taken as 100% (Figure 3A). The total mitochondrial NADH pool was estimated as a difference in arbitrary (arb) units between minimum fluorescence (after FCCP application) and maximum autofluorescence (after NaCN) (figure 3A). The basal redox level in L2122 (31.5±2.8%; n = 44; p<0.05), L2123 (27.8±1.8%; n = 37; p<0.001), L2124 (28.9±2.6%; n = 29; p<0.001) and L1703 fibroblasts (23.5±1.9%; n = 33; p<0.001) was significantly more oxidised compared to control C3 (62.1±3.9%, n = 41) and L2132 (54.6±5.2%; n = 28; Figure 3A–C). Interestingly, the cells with lowest Δψ_m_ - L2126 showed the highest redox index (78.9±6.4%, n = 47; Figure 3C). Provision of additional substrate - 5 mM glutamate, induced a significant increase of NADH in L2122, L2123, L2124 and L1703 cells (Figure 3B, C). The total mitochondrial pool of NADH was significantly lower in cells with PINK1 mutations, from 75.6±4.7% of control, p<0.05 in L2122 to 39.5±2.8% of control, p<0.001 in L1703 (Figure 3D).

**Figure 4:**
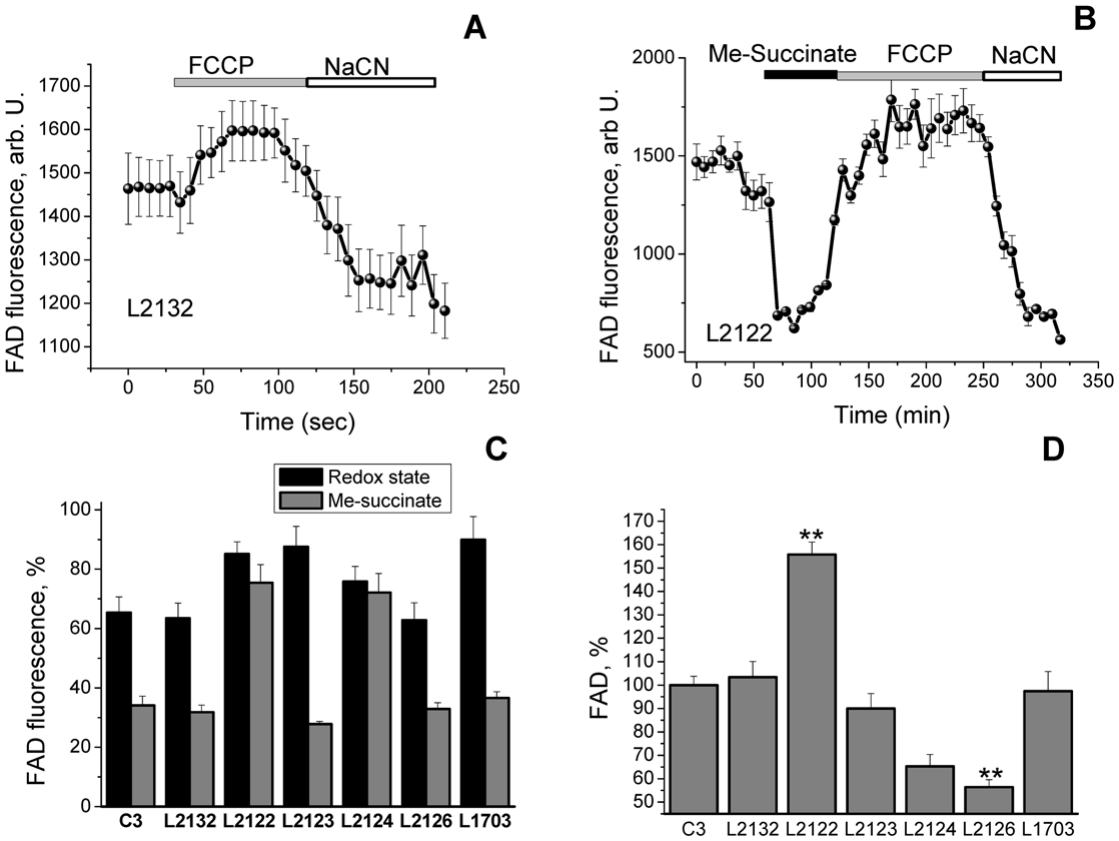
Level of FAD autofluorescence in fibroblasts with PINK1 mutations and control cells. Quantification of the %age change in FAD^++^ fluorescence: 100 is response to FCCP and 0% is response to cyanide, averaged traces for control L2132 and fibroblasts with PINK1 mutation L2122 presented in A–B. C-Fibroblasts with PINK1 mutations have increased FAD^++^ fluorescence than controls. This can be reversed by application of 5 mM Me-succinate to PINK1 mutated cells. D- Values of mitochondrial FAD autofluorescence.

The redox state (FAD) and mitochondrial level of flavoproteins was estimated in a similar way to NADH (only maximal oxidation with FCCP was taken as 100%, and maximal reduction with NaCN as 0%, Figure 4A). The FAD^++^ based redox state in L2122 (85.1±4.1%; n = 44; p<0.05), L2123 (87.6±6.8%, n = 37; p<0.05), L2124 (75.8±5.1%, n = 29) and L1703 (89.9±7.8%, n = 33; p<0.001) were significantly more oxidised compare to control C3 (65.4±5.3%, n = 41) and L2132 cells (63.5±5.1%, n = 28; Figure 4 B–C). Addition of substrate for complex II (5 mM me-succinate) normalized the redox level in L2122 and L2124 fibroblasts (Figure 4B–C). Provision of L2123, L2126 and L1703 fibroblasts with methyl succinate also reduced their redox level to control values (Figure 4 B–C). The level of the mitochondrial pool of flavoproteins involved in respiration was 1.55-fold higher in L2122 fibroblasts compare to control (p<0.001; figure 4D) and lower in L2126 (56.4±3.2% of control; p<0.001; Figure 4D). Thus, although L2122 fibroblasts have the same mild substrate deprivation as other PINK1 mutated cells, higher activity of the complex II allows them to maintain high membrane potential.

**Figure 5:**
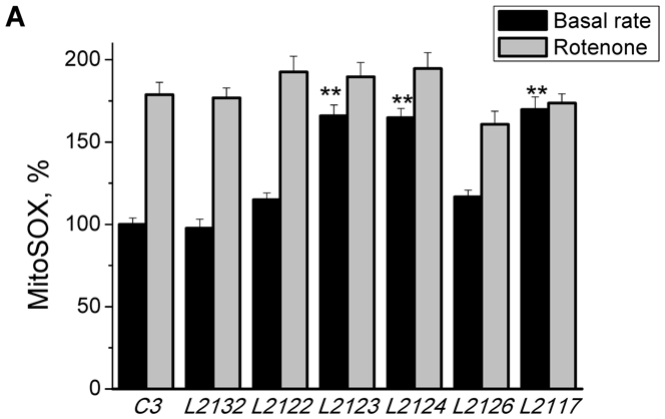
Increase in mitochondrial ROS production in fibroblasts with PINK1 mutations. Fibroblasts with PINK1 mutations displayed a higher basal rate of increase in Mitosox fluorescence, demonstrating a higher basal production of ROS compared to controls. Inhibition of complex I with 5 µM rotenone induced a significant increase in ROS production in control fibroblasts but only a small increase in ROS production in fibroblasts with PINK1 mutation. Histogram demonstrates %age values of rate of Mitosox fluorescence compared to 100% for control (C3) fibroblasts.

All the patient cell lines with *PINK1* mutations showed an increased basal rate of reactive oxygen species generation in mitochondria (figure 5A). The highest rates were observed in L2123 (165.9±6.7 of control rate; n = 37; p<0.05), L2124 (164.8±5.6%; n = 67; p<0.05) and L1703 (169.7±7.8%, n = 56; p<0.001). The application of 5 µM rotenone greatly stimulated ROS production in control cells (1.78-fold increase) but not substantially in *PINK1* mutated cells (L2123, L2124, L1703), although the increases in L2122 and L2126 were statistically significant (figure 5A). This suggests that mitochondrial ROS production in the cells with mutations in *PINK1* is already activated at basal levels due to the limited availability of complex I-linked substrates.

PINK1 knockout has been shown to result in inhibition of the mitochondrial Na^+^/Ca^2+^-exchanger and mitochondrial calcium overload [7]. Even a physiological calcium stimulus induced further overload of mitochondria with calcium and induced permeability transition pore (PTP) opening and a fall in Δψ_m_. Induction of the calcium signal in fibroblasts by 100 µM ATP (which stimulates purinoreceptors and the release of calcium from the endoplasmic reticulum) did not initiate changes in mitochondrial membrane potential of control (C3, L2132) or mutant (L2122, L2123, L2124, L2126 and L1703) cells (n = 3 experiments; data not shown). This suggests that physiological levels of calcium stimulation may not be sufficient to initiate pathological PTP opening in fibroblasts.

**Figure 6:**
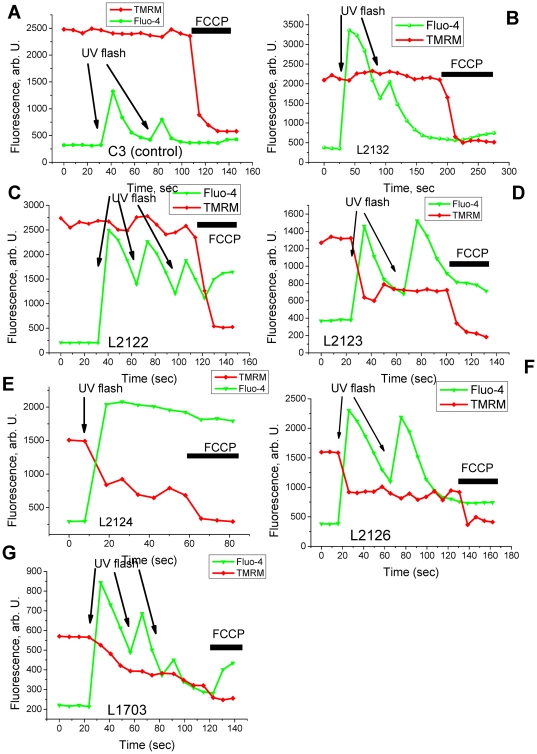
A rise in [Ca^2+^]_c_ induces mitochondrial depolarisation in fibroblasts with PINK1 mutations. Arrows mark UV-induced flash photolysis of cells loaded with Ca-NP-EGTA, fluo-4 and TMRM. In control C3 (A), L2132 (B) and in fibroblast with L2122 mutation (C) demonstrated an increase in [Ca^2+^]c in response to flash photolysis, with no change in Δψ_m_. In fibroblasts with PINK1 mutations (L2123 –D; L2124 – E; L2126 – F; and L1703-G), flash photolysis induced an increase in [Ca^2+^]_c_ with profound depolarisation of the mitochondria.

We employed the technique of UV flash photolysis of cells incubated with caged calcium (Ca-NP-EGTA) in order to generate a standard calcium signal free from variations in calcium influx/release. UV induced flash photolysis produced a rapid [Ca ^2+^]_c_ increase in control fibroblasts C3 (n = 18) and L2132 (n = 32) with no associated change in Δψ_m_ (figure 6A–B). L2122 fibroblasts showed no mitochondrial depolarisation in response to [Ca ^2+^]_c_ elevation (figure 6C). However, the same stimulus in fibroblasts with PINK1 mutations L2123 (n = 23; figure 6 D), L2124 (n = 19; figure 6E), L2126 (n = 21; figure 6F) and L1703 (n = 18; figure 6G) resulted in profound mitochondrial depolarisation. Thus, the enhanced mitochondrial membrane potential of L2122 and the lower ROS production in L2122 cells compared to other PINK1 mutation lines, increased the threshold for PTP opening and fall in Δψ_m_.

Mutations in the gene for PINK1 are a cause of autosomal recessive Parkinson's disease. PINK1 is a mitochondrial protein and recent studies have indicated that it plays a significant role in mitochondrial function and calcium homeostasis in particular [7]. These observations were derived from the use of cell culture knockouts including primary neuronal cultures from transgenic mouse models of PINK1 deficiency. Previous studies using PINK1 patient fibroblasts have shown defects of oxidative phosphorylation and the electron transport chain, and oxidative stress [9]–[11]. In this study we have for the first time studied PINK1 mitochondrial pathophysiology with single cell analysis of cultured cells derived from patients with PD and PINK1 mutations, in order to dissect the down-stream consequences on mitochondrial function. These cells represent an important model system as they enable the study of the biochemical effects of PINK1 mutations in intact cells devoid of any manipulation of the PINK1 gene, and in the presence of the host patient genomic background.

The *PINK1* mutant patient cells manifested multiple mitochondrial defects that paralleled those identified in the *PINK1* knockout models described above. *PINK1* knockdown by siRNA in human neuroblastoma cells was associated with reduction in glucose uptake at the plasma membrane [23] and with a reduction in mitochondrial DNA levels, decreased ATP synthesis and respiratory chain activity due to substrate restriction (low glucose), an increase in oxidative stress and abnormal calcium handling [7], [8]. Growth of PINK1 mutant cells in galactose caused mitochondrial fragmentation [11].

We have found in single fibroblasts that pathological mutations in *PINK1* (L1703, L 2123, L2126) cause a significant reduction in mitochondrial membrane potential and altered redox state (NADH level). As in the *PINK1* knockouts [7], [22], the PINK1 mutant fibroblast mitochondria switch from the production of ATP, to the consumption of ATP by the F1F0-ATPase in order to maintain their Δψ_m_. Furthermore, as in PINK1 knockouts, this phenomenon in L1703, L 2123, L2124 and L2126 mutants could be reversed by the provision of additional respiratory chain substrates: the increase in respiration in the presence of additional pyruvate resulted in a concomitant switch in the mechanism of Δψ_m_ maintenance from hydrolysis of ATP to production of ATP. The limitation in mitochondrial substrates seems to be a key point in PINK1 pathology. The increased activity in complex II (measured as FAD+ autofluorescence) in L2122 fibroblasts compensates for low NADH levels and leads to higher mitochondrial membrane potential, and protection against calcium induced PTP opening.

However, there were differences between the fibroblasts and neuronal models; we found significantly less cytosolic reactive oxygen species (ROS) production in *PINK1* mutant fibroblasts compared to *PINK1* knockout neurons that may be explained by a difference in NADPH oxidase activity. This would fit with our previous observations of normal glutathione levels and no increase in cytosolic oxidation in *PINK1* mutant fibroblast cultures [11]. *PINK1* mutant cells with low Δψ_m_ (L1703, L 2123, L2124 and L2126) were vulnerable to calcium stimulation and produced PTP opening (Figure 6). Furthermore, enhanced physiological calcium stimulation in *PINK1* knockout neurons lead to mitochondrial PTP opening while the calcium stimulus in *PINK1* mutant fibroblasts did not show mitochondrial depolarization. Fibroblast and neuronal cells are known to differ in their regulation of calcium homeostasis and in the role of mitochondria in calcium buffering [24]. Importantly, using artificial calcium stimulation (flash photolysis) in fibroblasts, we found that this induced opening of the PTP in cells with pathological mutations (figure 6). Our experimental conditions have therefore allowed us to demonstrate a defect in mitochondrial calcium regulation in these mutant cells that cannot be visualized with physiological stimulation.

Interestingly, the patient with the L2122 mutation had elevated Δψ_m_ and lower ROS production and so an increased threshold for PTP opening and fall in Δψ_m_ compared to other PINK1 mutation lines. This patient also had the latest age of onset for PD (age 61 y) compare to the other *PINK1* mutant PD patients. Although L2122 patient cells had the same mild substrate deprivation as other PINK1 mutated cells, the higher activity of complex II allowed the L2122 cells to maintain high membrane potential.

These studies have demonstrated that fibroblasts from PD patients with *PINK1* mutations exhibit very similar bio-energetic mitochondrial abnormalities to knockdown cell models. These patient cells also show that there is substrate dependent limitation on ATP synthesis that can be overcome with substrate replacement or normalization of glucose uptake. This may offer an interesting opportunity to explore in terms of disease modifying therapy in these patients, for which there is some precedent in patients with primary mitochondrial encephalomyopathies [25]. These data also indicate that patients with different PINK1 mutations, but affecting the same domain, can show different biochemical phenotypes. It is notable that the patient with the mildest defect of mitochondrial biochemistry (L2122) had the latest onset of disease. Whether this observation indicates a valid correlation with disease severity will require further work in additional patients.

The defects of mitochondrial function in PINK1 mutant patient cells described here have relevance to the part that PINK1 and parkin play in the removal of mitochondria by mitophagy [26]. It has recently been demonstrated in Drosophila and in mammalian cells that parkin ubiquitinates mitofusins 1 and 2, and this function is PINK1 dependent [16], [27]. This has also recently been demonstrated in fibroblasts from PD patients with PINK1 mutations [28]. Knockdown of *PINK1* causes mitochondrial dysfunction and accumulation of defective mitochondria. Overall cellular mitochondrial function can be restored by parkin overexpression and restoration of mitophagy pathways [16]. Thus, the abnormalities of energy metabolism and oxidative stress observed in our *PINK1* mutant patient cells will have a deleterious synergy with the parallel impairment of mitophagy, leading to the accumulation of defective mitochondria with the consequent impairment of ATP synthesis and increased free radical production. Such effects are likely to contribute to dopaminergic neuronal cell dysfunction and death.

## Methods

### Patients

Five patients in this study were diagnosed with PD and their clinical details have been presented previously [29]. The mutations in PINK1 and the basic clinical characteristics are summarized in Table 1. In these studies, patient 2132 served as a negative control i.e. a family member with no mutation and no disease (at age 35 years). A further control fibroblast line was used from an age-matched control at the same passage number as the *PINK1* mutant cells. All analyses were performed and patient samples obtained with the approval of the local ethics committee [University of Lubeck] and the written informed consent of the subjects involved.

**Table 1:** Clinical and biochemical characteristics of samples studied.

code	mutation gene Lab	mutation status lab	age at examination	disease duration	UPDRS II/III	Δψ_m_	Oligomycin	Redox level
L-1703	c.509C>G	hom.	68	38	missing	reduced	fall in Δψm	23.5±1.9
L-2122	c.1366C>T	hom.	67	6	33	increased	no change	31.5±2.8
L-2123	c.1366C>T	hom.	69	30	30	reduced	fall in Δψm	27.8±1.8
L-2124	c.1366C>T	hom.	60	7	36	reduced	fall in Δψm*	28.9±2.6
L-2126	c.1366C>T	hom.	71	24	38	reduced*	no change	78.9±6.4*
L-2132	none	no mutation found	35	N/A	0	normal	no change	54.6±5.2
C3	none	none		N/A	0	normal	no change	62.1±3.9

### Cell culture

Patient and control cells were cultured as previously described [11].

### Loading of cells

For measurements of [Ca^2+^]_c_ and Δψ_m_, cells were loaded for 30 min at room temperature with fluo-4 AM in combination with 25 nM tetramethylrhodamine methylester (TMRM) and 0.005% pluronic acid in a HEPES-buffered salt solution (HBSS) composed of (mM): 156 NaCl, 3 KCl, 2MgSO_4_, 1.25 KH_2_PO_4_, 2 CaCl_2_, 10 glucose and 10 HEPES; pH adjusted to 7.35 with NaOH. For flash photolysis experiments, caged Ca^2+^, 10 µM *o*-nitrophenyl EGTA, AM (NP-EGTA, AM) was loaded at the same time as the other indicators.

For measurements of Δψ_m_, cells were loaded with 25 nM TMRM for 30 mins at room temperature and the dye was present during the experiment. TMRM is used in the redistribution mode to assess Δψ_m_, and therefore a reduction in TMRM fluorescence represents Δψ_m_ depolarisation.

For measurement of mitochondrial ROS production, cells were pre-incubated with MitoSOX (5 µM, Molecular Probes, Eugene, OR) for 10 mins at room temperature.

### Imaging of [Ca^2+^]_c_ and Δψ_m_, ROS and autofluorescence of NADH and FAD^++^

Confocal images were obtained using a Zeiss 510 uv-vis CLSM equipped with a META detection system and a 40× oil immersion objective. The 488 nm Argon laser line was used to excite fluo-4 fluorescence which was measured using a bandpass filter from 505–550 nm. Illumination intensity was kept to a minimum (at 0.1–0.2% of laser output) to avoid phototoxicity and the pinhole set to give an optical slice of ∼2 µm. TMRM and MitoSOX were excited using the 543 nm laser line and fluorescence measured using a 560 nm longpass filter. NADH autofluorescence was excited at 351 and measured at 410 nm. 2-NDBG was excited at 458 nm, and fluorescence was measured at 520 nm. All data presented were obtained from at least 5 coverslips and 2–3 different cell preparations.

### Statistical analysis

Statistical analysis and exponential curve fitting were performed using Origin 8 (Microcal Software Inc., Northampton, MA) software. Results are expressed as means ± standard error of the mean (S.E.M.).
